# Effectiveness of a Novel Integrative Online Treatment for Depression (Deprexis): Randomized Controlled Trial

**DOI:** 10.2196/jmir.1151

**Published:** 2009-05-11

**Authors:** Björn Meyer, Thomas Berger, Franz Caspar, Christopher G Beevers, Gerhard Andersson, Mario Weiss

**Affiliations:** ^7^Ashridge Business SchoolBerkhamstedUnited Kingdom; ^6^Department of Clinical NeurosciencePsychiatry SectionKarolinska InstituteStockholmSweden; ^5^Department of Behavioural Sciences and LearningSwedish Institute for Disability ResearchLinköping UniversityLinköpingSweden; ^4^Department of PsychologyUniversity of TexasAustinTXUSA; ^3^Department of Clinical Psychology and PsychotherapyUniversity BerneBerneSwitzerland; ^2^GAIA AGHamburgGermany; ^1^Department of Psychosomatic Medicine and PsychotherapyUniversity Medical Center Hamburg-EppendorfHamburgGermany

## Abstract

**Background:**

Depression is associated with immense suffering and costs, and many patients receive inadequate care, often because of the limited availability of treatment. Web-based treatments may play an increasingly important role in closing this gap between demand and supply. We developed the integrative, Web-based program *Deprexis,* which covers therapeutic approaches such as behavioral activation, cognitive restructuring, mindfulness/acceptance exercises, and social skills training.

**Objective:**

To evaluate the effectiveness of the Web-based intervention in a randomized controlled trial.

**Methods:**

There were 396 adults recruited via Internet depression forums in Germany, and they were randomly assigned in an 80:20 weighted randomization sequence to either 9 weeks of immediate-program-access as an add-on to treatment-as-usual (N = 320), or to a 9-week delayed-access plus treatment-as-usual condition (N = 76). At pre- and post-treatment and 6-month follow-up, we measured depression (Beck Depression Inventory) as the primary outcome measure and social functioning (Work and Social Adjustment Scale) as the secondary outcome measure. Completer analyses and intention-to-treat analyses were performed.

**Results:**

Of 396 participants, 216 (55%) completed the post-measurement 9 weeks later. Available case analyses revealed a significant reduction in depression severity (BDI), Cohen’s *d* = .64 (CI 95% = 0.33 - 0.94), and significant improvement in social functioning (WSA), Cohen’s *d* = .64, 95% (CI 95% = 0.33 - 0.95). These improvements were maintained at 6-month follow-up. Intention-to-treat analyses confirmed significant effects on depression and social functioning improvements (BDI: Cohen’s *d* = .30, CI 95% = 0.05 - 0.55; WSA: Cohen’s *d* = .36*,* CI 95% = 0.10 - 0.61). Moreover, a much higher percentage of patients in the intervention group experienced a significant reduction of depression symptoms (BDI: odds ratio [OR] = 6.8, CI 95% = 2.90 - 18.19) and recovered more often (OR = 17.3, 95% CI 2.3 - 130). More than 80% of the users felt subjectively that the program had been helpful.

**Conclusions:**

This integrative, Web-based intervention was effective in reducing symptoms of depression and in improving social functioning. Findings suggest that the program could serve as an adjunctive or stand-alone treatment tool for patients suffering from symptoms of depression.

**Trial Registration:**

International Standard Randomized Controlled Trial Number (ISRCTN): 64953693; http://www.controlled-trials.com/ISRCTN64953693/64953693 (Archived by WebCite at http://www.webcitation.org/5ggzvTJPD)

## Introduction

Depression is associated with immense personal suffering and—due to treatment expenses and lost productivity—with high costs to the individual and society [1-3]. Despite the enormous burden imposed by depression, and even though depression is clearly treatable [4], many sufferers still receive inadequate treatment or no treatment at all [5-8]. For example, it has been estimated that only 10% of the 4 million people who suffer annually from depression are treated adequately in a well developed health care system such as the one established in Germany [9]. In other countries, similar problems are evident. For example, in the early 2000s, fewer than 25% of adults with major depressive disorders in the US received the recommended appropriate treatment [10]. The World Health Organization has estimated that during a 12-month period, about 14 million depressed individuals in Europe and 20 million in North and South America (combined) went untreated [11].

Many depressed patients who could benefit from treatment also remain on waiting lists for a long time or do not engage with treatment due to geographical inaccessibility, prohibitive costs, or other reasons, such as a preference for self-help [8]. The evidence shows that depressed patients who remain on waiting lists continue to report high levels of distress, even over many months [12].

What can be done to help more of these patients quickly and efficiently? In the UK, experts have recommended the training of 10,000 new therapists and the creation of new treatment delivery systems [13]. Similarly, a workgroup commissioned by the US National Institute of Mental Health [14] has recommended the development of innovative treatments that can be delivered at low costs to large populations. Specifically, the workgroup noted that “the Internet affords the opportunity to make psychosocial interventions available to large segments of the public. Interventions can be delivered programmatically and reliably, greatly extending the numbers and types of people who can be reached with services” [14] (page 623). In recent years, Web-based approaches have been increasingly used and it has been repeatedly shown that Internet-delivered treatments may be an effective and inexpensive alternative to traditional treatments [15-17]. Most of the existing Internet-based depression treatments are based on cognitive-behavioral principles, although other modalities, such as problem-solving therapy, appear promising as well [18].

The purpose of the project described here was to develop a novel, integrative program that could be delivered via the Internet to reduce symptoms of depression. The name *Deprexis* was chosen because it expresses which symptoms are targeted (ie, depression), and it conveys the idea that active practice is an inherent part of the treatment. The word is a combination of depression and *praxis*—a word of Greek and Latin origin denoting *deed* or *action*. The aim of this paper is to describe an initial study of its effectiveness.

## Methods

### Recruitment of Participants

The study was conducted between February of 2007 and June of 2008. Participants were recruited via advertisements posted on the Internet (eg, by posting brief notices on depression-related Internet forums in Germany, given the permission of the forum administrators). Upon establishing contact via email, potential participants received a detailed response email describing the project and inviting them to complete a set of online questionnaires. The email also informed potential participants that the program was not intended to replace psychotherapy or medical treatment and did not entail personal interactions with any treatment provider. Additionally, it explained that participants would be randomly assigned to one of two conditions: 9 weeks of access to an online self-help program or 9 weeks in a waitlist/delayed-access condition. Only those who provided consent, were above the age of 18, and completed at least half of the baseline depression questionnaire were included in the study. Similar to some previous studies in this area (eg, Warmerdam et al [19]) no other inclusion or exclusion criteria were used. The study was approved by an internal review board (IRB) in Frankfurt/Main (Hesse Ministry of Health, Germany).

### Intervention

**Figure 1:**
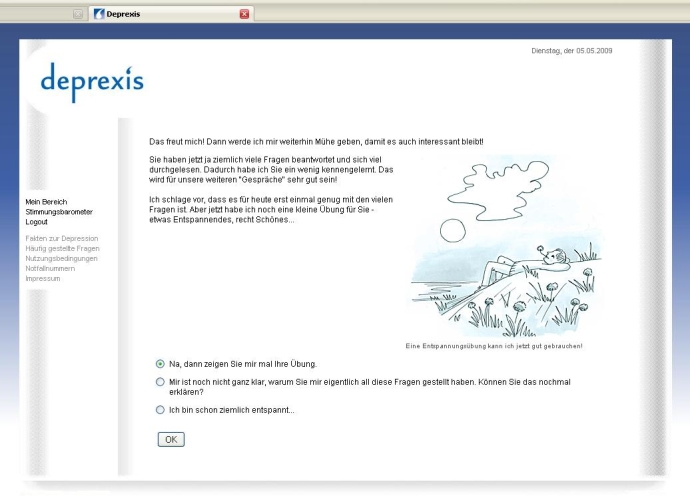
Example screenshot (see Multimedia Appendix 1 for additional examples)

The Web-based intervention consists of 10 content modules representing different psychotherapeutic approaches, plus one introductory and one summary module, each of which can be completed in 10 to 60 minutes, depending on the user’s reading speed, interest, motivation, and individual path through the program (see Figure 1 and Multimedia Appendix 1 for screenshots). Modules are organized as simulated dialogues in which the program explains and illustrates concepts and techniques, engages the user in exercises, and continuously asks users to respond by selecting from response options. Subsequent content is then tailored to the users’ responses, resulting in a simulated conversational flow. All modules are accompanied by illustrations (eg, drawings, photographs, flash animations). The program version that was evaluated in this study did not include audio or video features in order to increase accessibility by reducing the requirements for broad bandwidth and specialized hardware or software.

The modules cover a variety of therapeutic content that is broadly consistent with a cognitive-behavioral perspective, although the program is not restricted to one CBT manual. Instead, an effort was made to design the program as an integrative treatment tool that provides a variety of relevant therapeutic approaches and fits within the broad array of contemporary CBT. The modules’ theoretical rationale and content draws from theories like (1) Behavioral Activation, (2) Cognitive Modification, (3) Mindfulness and Acceptance, (4) Interpersonal Skills, (5) Relaxation, Physical Exercise and Lifestyle Modification, (6) Problem Solving, (7) Childhood Experiences and Early Schemas, (8) Positive Psychology Interventions, (9) Dreamwork and Emotion-Focused Interventions, and (10) Psychoeducation. Each is explained in turn below.

#### Behavioral Activation and Cognitive Modification

There is strong evidence that CBT techniques such as cognitive restructuring (eg, identifying and refuting unhelpful automatic thoughts, recognizing cognitive distortions, etc) and behavioral activation (eg, scheduling potentially enjoyable activities) are effective in the treatment of depression [4,20,21], so their inclusion in Web-based programs appears justified. Some controversy remains, however, with regard to the necessity of the cognitive elements of CBT packages. In some studies, behavioral activation alone has been as effective as, or even outperformed, the more cognitive CBT interventions [22-24]. In line with most CBT packages for depression, one *Deprexis* module was designed with a focus on behavioral activation (BA) and another with a focus on cognitive restructuring.

The BA module incorporates standard BA principles and procedures, as described in existing manuals (eg, Lejuez et al [25] and Martell et al [26]), but also contains modifications. For instance, users are encouraged to schedule activities that have the potential to satisfy basic psychological needs: the needs for social relatedness, autonomy, competence, self-esteem, and hedonic enjoyment. This need-satisfaction aspect is not a traditional element of BA but is a key feature of other treatments that have garnered empirical support, such as Grawe’s integrative therapy (a treatment that is well-known and widely used in German-speaking countries) [27,28].

The cognitive restructuring module incorporates standard cognitive intervention elements, as described in existing manuals [29-31], but it also contains modifications to adapt these approaches to the format and style of the program. A main emphasis in the cognitive modification module is on the mood-determining role of automatic thoughts; on the interaction between thoughts, emotions, overt behavior, and environmental events (ie, reciprocal determinism [32]; and on simple techniques that can be used to challenge or refute unhelpful automatic thoughts or to develop a more distanced and accepting attitude towards unhelpful thoughts.

#### Mindfulness and Acceptance

One of the most notable trends in psychotherapy research over the past decade has been the development of mindfulness- and acceptance-based interventions for depression, anxiety, and related syndromes and disorders [33-35]. Treatments such as acceptance and commitment therapy (ACT) [35] and mindfulness-based cognitive therapy for depression (MBCT) [36] have demonstrated their merit in terms of enhancing the effectiveness of traditional treatments [34,37]. In the *Deprexis* program, an acceptance/mindfulness module was designed to engage patients with key principles of such approaches. Brief exercises illustrate the difficulty of suppressing unwanted thoughts and feelings, and the idea that unwelcome experiences can be calmly observed and willingly accepted is presented via stories, metaphors, images, and texts.

#### Interpersonal Skills

Problems in interpersonal adjustment are well-known antecedents and concomitants of depression [38,39], and interpersonal psychotherapy for depression (IPT) [40,41] is a strongly empirically supported treatment [4,20]. Therefore, the inclusion of an interpersonally-focused module appeared justified. In this module, the role of social and interpersonal adjustment in the etiology and maintenance of depression is explained, and a variety of suggestions are provided to help users improve their interpersonal functioning and satisfaction. Such suggestions include, for example, tips for improved verbal and nonverbal communication as well as guidelines for relationship-enhancing behavior (eg, responding to good news conveyed by partners with enthusiasm rather than passive disinterest or active hostility) [42].

#### Relaxation, Physical Exercise and Lifestyle Modification

Physical exercise and healthy lifestyle behavior (eg, consuming healthy foods) are regarded as useful elements of integrative depression treatments [43,44,45]. Relaxation exercises, such as imagery and repeated tension exercises (eg, Suinn [46]) may also play a useful role in depression treatments, particularly for patients suffering from anxiety symptoms, which are exceedingly common in depression [47]. Given this evidence, a module was developed with a focus on relaxation exercises and healthy lifestyle tips. For example, users are guided through imagery and breathing exercises to help reduce tension and increase relaxation.

#### Problem Solving

Evidence indicates that problem-solving interventions are effective in the treatment of depression (eg, Mynors-Wallis et al [48], Mynors-Wallis et al [49], and Nezu [50]). In such treatments, patients learn how to define problems in concrete rather than vague terms, set achievable goals, generate potential solutions, evaluate different solution options, implement the chosen solution, and evaluate outcomes with respect to the original problem. Such algorithms are typically practiced repeatedly so that patients can generalize them to a variety of life problems and improve their overall problem-solving skills. One module is devoted to teaching and demonstrating this problem-solving approach to cope with a variety of depression-related problems.

#### Childhood Experiences and Early Schemas

Many depressed patients attribute their depression to problematic childhood experiences [51], and those who do regard childhood adversity as causally related to their depression tend to be specifically motivated to address unresolved childhood issues in psychotherapy [52]. Moreover, there is evidence that adversity in childhood predisposes to depression in later life [53], which further points to the importance of including interventions that target memories and other sequelae of difficult childhood experiences. Such interventions have shown empirical promise; for example, Young and colleagues’ schema therapy places “much greater emphasis on exploring the childhood and adolescent origins of psychological problems” than traditional CBT [54]. In the *Deprexis* program, one module focuses on difficult childhood memories. For example, the program explains techniques such as expressive writing [55-57], forgiveness [58], and acceptance of difficult memories [35].

#### Positive Psychology Interventions

Positive psychology focuses on the scientific study of positive experiences such as happiness, well-being, life satisfaction, and optimal functioning. From its inception in the late 1990s, the movement has become an increasingly dynamic force within psychology, with regular conferences, a journal, and various handbooks testifying to its momentum [59]. In recent years, the application of positive psychology to depression treatment has also been explored. Seligman and colleagues [60], for example, reported that positive psychology interventions such as encouraging people to cultivate strengths, expressing gratitude, and savoring positive experiences can lead to lasting reductions in depressive symptoms. In the *Deprexis* program, one module focuses on positive psychology interventions, including savoring positive experiences and memories, satisfying basic needs [61], and cultivating strengths and talents.

#### Dreamwork and Emotion-Focused Interventions

Although working with dreams is not a standard ingredient of empirically supported depression therapies such as CBT, there is evidence that therapeutic work with dreams can be a useful and productive therapeutic element, especially for patients who hold positive attitudes towards such approaches [62,63]. Rather than offering interpretations regarding the symbolic meaning of dreams, modern approaches to dreamwork use dreams as vehicles for creative problem solving [62]. In the *Deprexis* program, a dream and emotion-focused module is included and offered to users who indicate that they hold positive attitudes towards such content. The dialogue explains basic techniques such as keeping a dream journal, rewriting problem-laden dreams with positive endings, brainstorming about the relationships between dream contents and real-life problems, and others (cf Morris [64]).

#### Psychoeducation

Psychoeducation is an important aspect of many empirically supported depression interventions (eg, CBT) [30,65]. Therefore, the *Deprexis* program also includes a module that explains basic descriptive aspects of depression. This includes, for example, a review of the diagnosis of major depression (as a brief, jargon-free summary), an overview of diathesis-stress models of depression (emphasizing the interaction between personal and environmental factors in depression), a section on biological and medical aspects of depression, and a synopsis of cultural aspects. This module is offered optionally, although psychoeducational elements are included throughout other modules as well. Furthermore, a review module is offered in which key ideas of other modules are briefly reviewed. Users are encouraged to repeat all modules as often as they wish after they have passed once through the module sequence.

### Design

In order to examine the effectiveness of the *Deprexis* program, a randomized controlled trial was conducted with help-seeking adults who reported symptoms of depression. It was hypothesized that, over the course of 9 weeks, program users would achieve greater reductions of depression symptoms than comparison participants in a delayed-access, treatment-as-usual (TAU) condition. Additionally, we hypothesized that the majority of users would evaluate the *Deprexis* program favorably and report that they benefitted from using it.

For the main hypothesis, a 2 x 2 (pre vs post by treatment vs waitlist-control condition) design was used. Participants completed baseline (T0) self-reports of depression severity and other variables online and were then assigned either to the immediate-treatment condition (9 weeks of access to the program) or to a waitlist/delayed-treatment condition, in which they received access to the program after waiting for 9 weeks. At the 9-week time-point (T1), participants were invited to complete online questionnaires to determine whether the immediate-treatment group had, indeed, improved to a greater degree than the waitlist/delayed access group.

For exploratory purposes, we also gathered follow-up data, beyond T1. The delayed-access group completed post-treatment questionnaires, which coincided with the 9-week follow-up data collection time-point of the immediate-treatment group (T2). This design enabled us to test whether any treatment effect that might be observed in the immediate-treatment group could be replicated among those in the delayed-treatment condition. The delayed-treatment group was also asked to complete 9-week follow-up questionnaires (T3), and both groups were invited to complete follow-up questionnaires 6 months after treatment termination (T4). The outcome variable of primary interest was depression severity, as measured by the Beck Depression Inventory (BDI). However, given the exploratory, open nature of this study, we did not limit our focus on patients with a bona fide diagnosis of a depressive disorder, and we regard the BDI as a measure of general distress, which correlates highly with depression as well as with other forms of emotional distress [12].

### Randomization

We used a weighted randomization procedure in which 80% were assigned to the immediate-treatment condition and 20% to the delayed-treatment condition. The purpose of this weighting was to ensure that a sufficiently large number of participants would take part in the treatment and would be able to provide feedback that could be used for further program development. An a priori power analysis indicated that, given this 4:1 weighted randomization strategy, at least 200 participants (immediate-treatment group: 160; delayed-treatment condition: 40) would be required to achieve a power level of > .80, assuming a medium effect size, (Cohen’s *d* = .50), with alpha set at .05 (two-tailed). The goal was to retain this number of participants at the T1 time-point, after those in the immediate-treatment condition had completed 9 weeks of program access, and those in the delayed-treatment condition had waited for program access for an equal duration.

Randomization was performed via a computer generated list of random numbers. After generating a list of 500 random numbers and sorting them by size, the highest 20% were marked to indicate that they referred to the control condition. The list was then resorted to its original order and newly enrolled participants were consecutively placed onto this list. If a new participant received a marked number, he or she was assigned to the control condition; otherwise, the new participant was assigned to the immediate-access condition. This procedure ensured that an 80:20 chance—but no predictable sequence—existed with regard to whether a new participant would be assigned to the immediate-access or the delayed-access condition.

### Measures

#### Beck Depression Inventory (BDI)

The BDI [66,67] is one of the most commonly used self-report measures of depression severity and has well established validity and reliability [68]. The German version of the BDI [67], which was used in this study, includes 21 items measuring symptoms such as hopelessness, irritability, guilt, feelings of being punished, fatigue, weight loss, and lack of interest. Cronbach alpha of .84 (T0) indicated good internal consistency of the BDI in this study. Because of ethical concerns, the suicidality item was dropped from the BDI, but this missing item-score was imputed from the remaining 20 items so that the sum scores are comparable to established 21-item BDI norms. The BDI was administered at each of the assessment time-points.

#### Work and Social Adjustment Scale

This 5-item questionnaire measures the extent to which the respondent’s depression interferes with his or her ability to perform various tasks of daily living, such as household chores, hobbies, or private leisure-time activities [69]. In the present study, internal consistency was excellent (Cronbach alpha = .83, T0). The WSA was scored on a 1-9 Likert-type response scale. The use of this questionnaire was exploratory in the present study because a translated (German) version was employed, which has not been validated in Germany so far. Given the face-valid nature of these items and high internal consistency, however, it seems likely that the translated WSA would still yield a useful estimate of depression-related psychosocial impairment. The WSA was also administered at all time-points.

#### Additional Questions: Program Acceptability and Subjective Benefit

A series of questions was administered to evaluate the extent to which participants felt they benefited personally from the program, liked or disliked the program, and would recommend the program to others. These questions are described in detail in the results section.

### Statistical Analyses

Preliminary descriptive statistics and correlational analyses were conducted to illuminate the associations between program usage (number of sessions completed) and changes in depression over time. To test the hypotheses that depression and social dysfunction scores would decrease as a consequence of program usage versus scores for those assigned to the control group, both intention-to-treat (ITT) and available-cases analyses were conducted. For the intention-to-treat analysis, we conducted mixed-model repeated measures ANOVA with time (pre-post) as a within-groups factor and treatment condition as a between-groups factor. Mixed-model repeated measures ANOVA uses all available data on each subject and does not involve the substitution of missing values. In addition, and as a comparison, a 2 x 2 repeated measures ANOVA was applied to a dataset in which pre-treatment data were carried forward for non-completers to replace missing values. A repeated-measures ANOVA with time as a within-subjects and group (immediate *Deprexis* use vs waitlist control) as a between-subjects independent variable was also used to analyze the available-cases. In addition to tests of statistical significance and computation of effect sizes, we also computed the clinical significance of the observed effects, using standardized procedures as described in detail below.

## Results

### Demographics, Response Rates, and Attrition

As summarized in Table 1, a total of 396 individuals was included in the study, of which 81% were assigned to the immediate-treatment condition and 19% to the delayed-treatment condition. Table 1 shows that the average age was around 35, with a range from 18 to 72. About ¾ of the sample were women, consistent with the well-documented predominance of women among depression sufferers. Many of the participants in this study were quite incapacitated in terms of symptom severity and social dysfunction. For example, slightly over half of the sample was currently unemployed, more than half reported currently being in treatment (medication and/or psychotherapy), and 85% stated they had been feeling depressed for several months (29%) or even several years (56%). There was no significant difference between the intervention and control group on any of the baseline variables, including baseline depression and social functioning (Table 2).

**Table 1:** Sample characteristics

	Immediate treatment group	Delayed treatment group	Total (combined) sample	*P* Value
**Time 0 (Baseline)**				
*N* (%)	320 (80.81%)	76 (19.19%)	396 (100%)	
Age (M, SD)	34.58 (11.53)	35.47 (11.98)	34.76 (11.60)	.55
Gender (% Female : % Male)	77 : 23	71 : 29	76 : 24	.30
% married or partnered	51%	58%	53%	.37
% completed univ degree	18%	20%	18%	.61
% currently unemployed	51%	56%	52%	.44
% previously treated for depression	66%	70%	67%	.54
% in current treatment for depression	58%	64%	59%	.32
% currently receiving psychotherapy-only vs medication-only vs both	13% vs 20% vs 23%	11% vs 31% vs 20%	13% vs 22% vs 22%	.14
**Time 1 (9 weeks)**				
*N* (%)	159 (74%)	57 (26%)	216 (100%)	
Age (M, SD)	34.89 (11.40)	35.25 (11.79)	34.99 (11.48)	.84
Gender (% Female : % Male)	78 : 22	70 : 30	76 : 24	.24
% married or partnered	54%	64%	57%	.16
% completed univ. degree	18%	20%	18%	.84
% currently unemployed	46%	57%	49%	.16
% previously treated for depression	67%	72%	70%	.50
% in current treatment for depression	60%	68%	62%	.34
% currently receiving psychotherapy-only vs medication-only vs both	16% vs 20% vs 22%	12% vs 31% vs 21%	15% vs 23% vs 22%	.36

**Table 2:** Descriptive statistics: depression and social functioning

	Immediate-treatment group M (SD), N	Delayed-treatment group M (SD), N	Mean comparisons and effect size (between-groups Cohen’s *d*)
**Depression (BDI)**			
T0 (baseline)	26.72 (9.86), 320	27.11 (8.98), 76	*t* (394) = .31, *P* = .76 (*d* = .04)
T1 (9 weeks)	19.87 (11.85), 159	27.15 (10.01), 57	*t* (214) = 4.14, *P* < .001 (*d* = .64)
T2 (18 weeks)	17.23 (11.85), 111	20.39 (12.92), 35	*t* (144) = 1.34, *P* = .18 (*d* = .25)
T3 (27 weeks)^a^		19.07 (15.32), 25	
T4 (6-months follow-up)	16.50 (12.93), 85	15.25 (14.80), 14	*t* (97) = -.33, *P* = .74 (*d* = .09)
**Social Dysfunction (WSA)**			
T0 (baseline)	5.66 (1.66), 315	5.89 (1.50), 75	*t* (388) = 1.10, *P* = .27 (*d* = .15)
T1 (9 weeks)	4.80 (2.14), 154	6.06 (1.42), 57	*t* (209) = 4.11, *P* < .001 (*d* = .64)
T2 (18 weeks)	4.48 (2.26), 109	4.65 (1.92), 34	*t* (141) = .40, *P* = .69 (*d* = .08)
T3 (27 weeks)^a^		4.86 (2.30), 24	
T4 (6-months follow-up)	4.10 (2.41), 83	4.07 (2.74), 12	*t* (93) = -.04, *P* = .97 (*d* = .01)

^a^At the T3 data-collection time-point, questionnaires were administered only to the delayed-treatment group, given that this constituted the 9-week post-treatment follow-up for that group.

**Figure 2:**
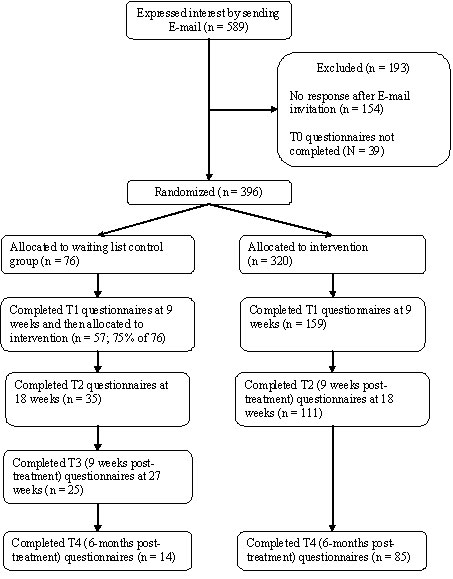
Participant flow

In terms of attrition, between one-third and half of the sample was lost from the study at each time-point (Figure 2). That is, from 396 participants who were initially randomized at T0, 216 (55%) completed the depression questionnaire 9 weeks later (T1). Similarly, of these 216 T1 participants, 146 (68%) completed the depression questionnaires at the 18-week time-point (T2). Of these participants, 99 (68%) were available for the 6-month follow-up data collection time-point (T4).

With regard to the post-assessment (T1), the response rate was higher in the control group (75%; n = 57) than in the intervention group (50%; n = 159; *P* < .001). However, there was no difference at post-assessment (T1) between the intervention and control group on any of the assessed client characteristics (Table 1).

**Figure 3:**
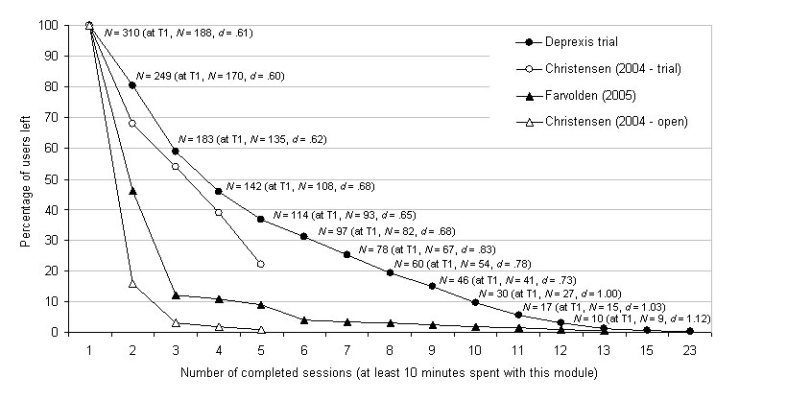
Program usage over time: comparison between Deprexis participants and similar studies (data from Eysenbach)

Figure 3 presents a graphic overview of program usage. These descriptive statistics are based on the entire sample, collapsing across the immediate-access and delayed-access groups. Comparisons between these groups are presented further below. Of the 396 participants who completed the initial T0 questionnaire and were randomized to conditions, 19 (4.8%) never logged on to the program again and can be considered pre-treatment drop-outs. Another 67 (16.9%) never completed a single session of at least 10 minutes duration and can be considered early drop-outs. The 86 drop-outs did not differ from the 310 actual program users in terms of baseline depression severity, social functioning, age, gender, self-reported depression chronicity, and current as well as past depression treatment (eg, medication, psychotherapy, or both).

Figure 3 shows that 310 users completed at least 1 session of more than 10 minutes over the course of the entire study. Of these 310 users, 249 (80.3%) completed at least 2 sessions of more than 10 minutes duration, 183 (59.0%) completed at least 3 such sessions, and only 2 users (0.6%) completed more than 13 sessions. It was possible to complete more than 12 sessions because each module could be repeated once or several times, depending on the user’s preference. Thus, there was no upper limit to the number of sessions a user could do. In practice though, as shown in Figure 3, the upper limit was 23—the number of sessions completed by a single user.

Figure 3 also presents comparison data from similar studies, as discussed in Eysenbach’s “Law of Attrition” article [70]. The figure shows that the attrition rate in the current study appeared favorable compared to previous studies in which no therapist support has been included.

Figure 3 also shows the number of users who were available for the post-treatment assessment time-point, grouped by the number of sessions completed. For example, of the 310 participants who completed at least 1 session, 188 (61%) completed the post-treatment assessment. Of the 60 who completed at least 8 sessions, though, 54 (90%) completed the post-treatment assessment. As one might expect, the more sessions users completed, the more likely they were to complete the post-treatment assessment. A very high correlation confirmed the impression of such a strong linear association between program usage and study compliance (*r* = .91, *P* < .001, N = 12, completion percentages derived from the values shown in Figure 3 were correlated with the number of sessions, from 1 to 12, shown on the x-axis).

Figure 3 also shows the pre-post treatment effect sizes (Cohen’s *d*) of depression improvement, as measured by the BDI, for users who completed different numbers of sessions. For example, the pre-post effect size for those 188 users who completed at least one session was .61. The pre-post effect size of those 78 users who completed at least 7 sessions, by contrast, was .83. Indeed, the correlation between number of sessions completed and effect size was also extremely high (*r* = .91, *P* < .001, N = 12). For this analysis, the effect sizes shown in Figure 3 were correlated with number of sessions shown on the x-axis, from session 1 to 12.

These strong associations suggested that users who engaged more often and intensively with the program were more likely to complete the follow-up assessment and to benefit from the program. These preliminary analyses do not answer the question, though, of whether differences exist in symptomatic and functional improvement between those in the treatment versus the waitlist group. The next section presents the relevant comparisons.

### Symptoms of Depression and Social Functioning

#### Intention-to-Treat Analyses

As shown in Table 1, Figure 2, and Figure 3, and as discussed above, attrition was a considerable problem in this study: between 30% and 50% of participants were lost between any two assessment time-points, and fewer than 50% of the users completed more than 3 sessions. Several questions arise, therefore: what happened to those who chose not to continue the program and not to complete the post-treatment and follow-up questionnaires? Would analyses based on the completer sample exaggerate true effect sizes?

To respond to these concerns, we conducted intention-to-treat (ITT) analyses in two ways. First, we analyzed the data by using mixed-model repeated measures ANOVA with time (pre-post) as a within-groups factor and treatment condition as a between-groups factor. Mixed-model repeated measures ANOVA uses all available data on each subject and does not involve the substitution of missing values [71,72]. Second, and as a comparison, analyses were undertaken using a dataset in which the missing T1 data for those participants who did not complete the T1 questionnaires was set at their baseline (T0) level. This last observation carried forward (LOCF) approach assumes that, of the 320 participants who were assigned to immediate treatment at T0, the 161 who did not complete T1 questionnaires did not improve at all. The LOCF-dataset was analyzed with a 2 x 2 repeated measures ANOVA with time as the within-group factor and treatment as the between-group factor.

In the mixed-model repeated measures procedure, relationships between the observations at pre- and post-assessment were modeled as an unstructured covariance matrix. With regard to the BDI, a significant interaction between treatment condition and time (T0 vs T1) was found (F_1,219.7_ = 19.2, *P* < .001). Based on estimated marginal means, the immediate-treatment group improved 5.4 BDI points (from 26.72 to 21.30), which corresponded to a pre-post effect size of *d* = 0.58. By using the estimates from the mixed-model, the between-groups effect size was at *d* = 0.65.

Using the LOCF-dataset, the 2 x 2 repeated measures ANOVA showed a significant interaction between treatment condition and time (T0 vs T1) in the prediction of BDI-scores, F_1,394_ = 10.12, *P* = .002. In this sample, there was a reduction of 3.11 BDI points between T0 and T1 among the 320 participants assigned to the immediate-treatment group (from 26.72 to 23.61, pre-post Cohen’s *d* = .29). This change was significant, paired-*t*_319_ = 7.20, *P* < .001. Among the 76 participants assigned to the delayed-treatment group, which did not have access to the program at this time, depression levels remained unchanged in this ITT sample (27.11 to 27.07, pre-post Cohen’s *d* = .00, paired-*t* = .05, *P* = .96). The between-groups effect size at T1, using this ITT sample, was Cohen’s *d* = .30.

Similar analyses were performed with the WSA. The mixed-model repeated measures ANOVA revealed a significant interaction between treatment condition and time (F_1,402.1_ = 7.7, *P* = .006). The within-groups effect size based on the estimated marginal means was at *d* = .47, the between-groups effect size at *d* = .63. Using the LOCF-dataset, the 2 x 2 repeated measures ANOVA showed a significant interaction, F_1,388_ = 6.98, *P* = .009. Whereas social dysfunction decreased slightly in the immediate-treatment group, paired-*t*_314_ = 4.15, *P* < .001, there was no significant change in the delayed-treatment group between T0 and T1, paired-*t*_74_ = -1.02, *P* = .31. The pre-post effect size in the immediate-treatment group was Cohen’s *d* = .17, and the between-group effect size at T1 was Cohen’s *d* = .36.

Overall, both analyses revealed clear evidence of reductions in depression and social dysfunction in response to the treatment. Results obtained using LOCF were less pronounced, suggesting that the LOCF-procedure produces more conservative estimates of effectiveness. However, mixed-model repeated measures ANOVA is more and more recognized as the preferred choice for the analysis of repeated measures data [71].

#### Available Case Analyses

Descriptive statistics for the BDI and the WSA at all time-points are shown in Table 2. The mean comparisons shown in the table are based on data from participants who actually completed the questionnaires at each time-point. Statistics for the intention-to-treat sample are discussed above.

Table 2 shows that, as predicted, using the program was associated with improvements in depression severity and social dysfunction, whereas not using the program was associated with no improvement. Consistent with the hypotheses, the immediate-treatment group scored significantly lower on depression and social dysfunction at T1, compared to the delayed-access group, but the respective values did not differ at any of the other time-points.

The between-group differences in depression and social dysfunction at T1 correspond to effect sizes of *d* = .64 on both measures.

A 2 x 2 repeated measures ANOVA with the BDI as dependent variable, time-point as a within-subjects independent variable (T0 vs T1), and treatment condition as a between-subjects independent variable was conducted. Only participants who completed questionnaires at both T0 and T1 were included in this analysis. This ANOVA showed a significant interaction and confirmed the main hypothesis, that depression levels would decrease more among those in the immediate-treatment rather than the delayed-treatment condition, F_1,214_ = 17.81, *P* < .001. There was a significant reduction in BDI-scores between T0 and T1 among those in the immediate-treatment group, paired-*t*_158_ = 7.87, *P* < .001 (pre-post Cohen’s *d* = .58), but no change in depression between T0 and T1 among those in the delayed-treatment group, paired-*t*_56_ = .05, *P* = .96 (pre-post Cohen’s *d* = .01).

Similarly, a significant interaction was found with the WSA as the dependent variable, F_1,206_ = 9.17, *P* = .004. Again, social dysfunction improved significantly between T0 and T1 among those in the immediate-treatment group, paired-*t*_151_ = 4.27, *P* < .001 (pre-post Cohen’s *d* = .33), but not among those in the delayed-treatment group, paired-*t*_55_ = -1.02, *P* = .31 (pre-post Cohen’s *d* = .12).

**Figure 4:**
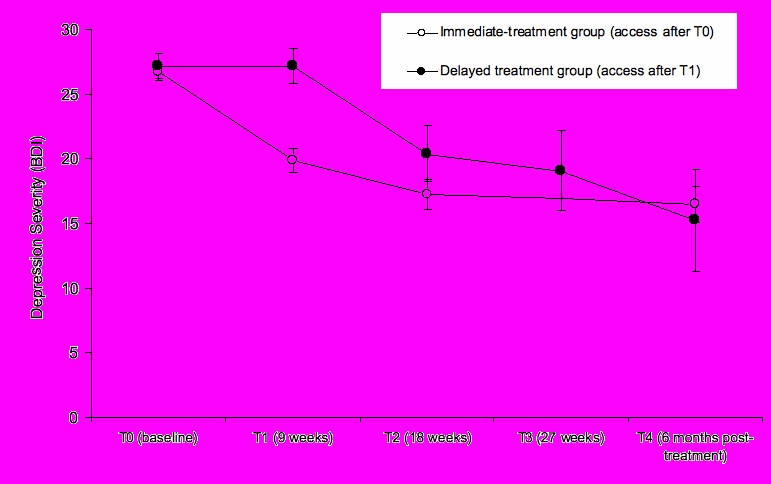
Depression severity over time: comparison between the immediate-treatment versus delayed treatment groups (data points are based on all participants who completed questionnaires at each respective time-point)

The course of depression symptoms is graphically depicted in Figure 4, which shows that, at baseline, depression severity was in the moderate-to-severe range in both groups. Note that the data points in Figure 4 are based on all participants who completed questionnaires at each respective time-point (eg, in the immediate-treatment group, N = 320 at T0, N = 159 at T1, et cetera, Table 2).

Once the treatment was received, there was a marked reduction of around 6 BDI-points in both the immediate-treatment (reduction by 6.26 points, on average, among those 159 participants in the immediate-treatment group who completed both the T0 and T1 BDI) and the delayed-treatment (reduction by 5.94 points, on average, among those 34 participants in the delayed-treatment group who completed both the T1 and T2 BDI) groups.

Among those in the immediate-treatment group, the reduction in depression severity in the 9 weeks following the treatment, between T1 and T2, was also significant, paired-*t*_88_ = 3.16, *P* = .002. After this, depression levels remained stable in the mild-to-moderate range, around 17, with no significant change between T2 and T4, paired-*t*_52_ = .40, *P* = .69. Among those in the delayed-treatment condition, there were no significant symptom changes after completion of the treatment (*P*s > .30). In this group, the marked change in depression also occurred in response to the treatment, and symptoms remained in the mild-to-moderate range at the 6-months follow-up time-point (Figure 4). The pre-post effect size for those 85 participants who completed the BDI at both T0 and at the 6-month follow-up (T4) was *d* = .74. For those 14 in the delayed-access group, the T0 - T4 effect size was *d* = .96.

#### Clinical Significance of Depression Changes

Data on clinically significant improvement as defined by Jacobson and Truax [73] are presented in Table 3. Following the recommendations of Seggar, Lambert, and Hasen [74], reliable change was defined as a move of at least 8.46 points on the BDI from pre-test to post-test (ie, from T0 to T1). Furthermore, a post-test score of below 14.29 needed to be achieved in order for the improvement to be considered clinically significant [74]. For these analyses, dropouts were not included. Also, only participants who exceeded the cut-off score of 14.29 at the T0 pre-test time-point were included in order to have a chance to move from a dysfunctional to a functional range.

**Table 3:** Data for the proportion of participants reaching the criteria of clinical significant improvement (recovered) or of reliable change (improved but not recovered)

	Immediate treatment (n = 138)		Control (n = 52)		
	%	n	%	n	χ^2^(1)
Recovered	25.4	35	1.9	1	19.08 (*P* < .001)
Improved but not recovered	16.7	23	7.7	4	
No reliable change	53.6	74	82.7	43	
Deteriorated	4.3	6	7.7	4	

As can be seen in Table 3, there were significant differences in terms of clinically significant improvement between the immediate-treatment and the waitlist/control group. About one-quarter of those assigned to the immediate-treatment condition showed large improvements in depression severity with post-treatment scores more in line with non-clinical than clinically depressed populations. Such improvements were extremely rare among those assigned to the waitlist/control group (occurring in only 1 out of 52 cases). Whereas 42.1% of those assigned to the immediate-treatment condition showed reliable improvement or recovery, this was true for less than 10% of those in the waitlist/control group.

The proportions of clinically significant improvement shown in Table 3 compare the immediate-treatment with the waitlist/control group. But what were the rates of improvement among those in the control group after they also received the treatment? Of the 31 participants with complete T2 data, 7 (22.6%) could be classified as recovered, 5 (16.1%) as improved but not recovered, 17 (54.8%) as not reliably changed, and 2 (6.5%) as deteriorated. Thus, even though the sample size was considerably smaller for this delayed-treatment group, the rates of improvement shown in Table 3 were closely replicated. It appears that about 40 of 100 program users will clearly benefit, with up to 25 of those achieving post-test scores in the recovered range. Of the 55 - 60 who do not benefit, the vast majority will simply show no clear change in either direction, and fewer than 5 of 100 can be expected to deteriorate.

### Subjective Benefit and Acceptance of the Program

Table 4 provides an overview of the questions that were asked to estimate participants’ subjective satisfaction with the program. Approximately 80% of the users were generally satisfied. For example, 83% gave the program a grade between 1 and 3 on a 1 - 6 scale (with 58% assigning a score of 1 or 2); 82% had the sense that the program had helped at least a little bit; 78% reported that the program had met or exceeded their expectations; 74% felt that the program’s tips and suggestions were as good or better than those given by human therapists; and 95% would recommend it to others suffering from mild depression (79% would recommend it to others with moderately severe depression and 42% to those with severe depression). Table 4 also shows that none of the participants felt that the program had harmed rather than helped them.

**Table 4:** Subjective benefit and user impressions

			Number and percentage of participants
Overall impression: How did you like the program, all in all? (1 - 6 scale, 1 = very good, 6 = seriously flawed)			
	Liked the program (1-3)		164 (83%)
	Did not like the program (4-6)		34 (17%)
Subjective benefit: Do you have the sense that the program helped you?			
	Helped me a lot		28 (14%)
	Helped me a little		139 (68%)
	Did not help		36 (18%)
	Did more harm than help		0 (0%)
User satisfaction: Did the program meet your expectations?			
	Positively surprised: The program exceeded my expectations		36 (18%)
	Satisfied: The program met my expectations		119 (60%)
	Disappointed: The program did not meet my expectations		42 (21%)
Quality of content: How would you rate the program’s tips and suggestions compared to a “real” (human) psychotherapist?			
	Content was better than human therapist		31 (16%)
	Content was about as good as human therapist		111 (58%)
	Content was worse than human therapist		48 (25%)
Recommendations: Would you recommend the program to others...			
	...who are suffering from mild depression?		
		- would definitely not recommend it	5 (3%)
		- would probably not recommend it	4 (2%)
		- would recommend it with reservations	32 (16%)
		- would definitely recommend it	156 (79%)
	...who are suffering from moderately severe depression?		
		- would definitely not recommend it	9 (5%)
		- would probably not recommend it	31 (16%)
		- would recommend it with reservations	92 (47%)
		- would definitely recommend it	63 (32%)
	...who are suffering from severe depression?		
		- would definitely not recommend it	56 (29%)
		- would probably not recommend it	57 (29%)
		- would recommend it with reservations	60 (31%)
		- would definitely recommend it	21 (11%)

## Discussion

In the randomized controlled trial described here, adults who used the *Deprexis* program improved, on average, by about 6 BDI points, whereas those in a delayed-access control condition did not improve at all during the waiting period. On average, participants initially reported being moderately to severely depressed, but by the end of treatment, only mildly to moderately depressed. Among those who completed the pre- and post-treatment questionnaires, the treatment effect corresponded to an effect size of .64 (post-treatment between-groups comparison) and was replicated when the waitlist control group also received access to the program. In the ITT analyses, significant treatment effects were also observed, although the effect sizes were weaker (eg, *d* = 30 for the between-groups effect at T1).

The gains in depression improvement were maintained over a follow-up period of 6 months, and positive changes were also demonstrated in terms of social functioning. About one-quarter of the participants experienced clinically significant rates of depression improvement, such that they no longer reported being depressed after the treatment. Half of the participants did not report such improvements, although about 80% of the users subjectively felt that the program had been helpful. In sum, these findings strongly suggest that the *Deprexis* program can be a useful and effective treatment for help-seeking Internet users suffering from depression.

The findings from the study suggest that online programs for depression can work even in the absence of therapist support. These findings are consistent with previous evidence, which demonstrated the effectiveness of other online depression programs, such as the Australian MoodGym program or the US-American ODIN program [75-78]. Overall, then, a clear effect of online support for depression has been established [17], although unguided online depression programs tend to achieve relatively low effect sizes [15-17]. The present study tentatively suggests that online programs might work better if interactivity is emphasized and a wide range of treatment ingredients are included. Compliance and dropout still remain problematic, but it may be possible to increase adherence by providing participants with a clear deadline and scheduled follow-up appointments, even if these are automated.

A surprising observation in this study was that a large proportion of participants showed lasting positive effects even though they received only a small dosage of the treatment (ie, 4 sessions or fewer). This finding is actually consistent with previous research showing that many psychotherapy clients experience the majority of therapeutic gains within the first few sessions. Howard et al [79], for example, found that 41% of therapeutic gains typically occur within the first 4 sessions. Similarly, Kopta et al [80], found that 50% of patients achieve symptomatic recovery from depression after only 5 sessions. Barkham et al [81] as well as Stiles et al [82] also recently found that more than 70% of patients in routine psychotherapy who only attended fewer than 4 sessions achieved reliable and clinically significant improvement. Once they achieve a personal “good enough” level, many of these patients terminate treatment because the most pressing treatment goals have been achieved. In open-access Internet treatments, this possibility also seems plausible: many of those who dropped out after only a few sessions in this study may have done so because they felt that they had reached a “good enough” level or had received an adequate amount of help from the program. An alternative possibility is, of course, that many of these participants dropped out early because they did not find the program useful. Future research will be needed to disentangle and further understand these possibilities.

### Limitations

The results of the current study must be interpreted in light of several limitations. A major caveat in interpreting these results concerns the high attrition rate. Only about half of those who had completed the baseline questionnaires and entered the study also completed questionnaires 9 weeks later, at the post-treatment time-point for the immediate-access group. Furthermore, only about half of the users completed more than 3 sessions (Figure 3). Nevertheless, ITT analyses revealed significant treatment effects even when one assumes that all dropouts remained at their initial level of depression severity. Thus, it seems unlikely that the observed effects are spurious or due to the fact that non-improvers dropped out.

In this context, Eysenbach [70] also highlighted the finding that high attrition rates are actually expected when conducting open Internet trials without any therapist support (see also Andersson [83]). When participants can easily discontinue without adverse consequences, many of them will regularly do so. Future efforts in this area would be well advised to explore new methods to increase treatment engagement and adherence. For example, brief telephone-delivered interventions that are based on motivational interviewing might improve engagement and reduce attrition among depressed patients [84,85].

A second limitation of the study concerns the heterogeneous sample of users. Future investigations would benefit from studying more narrowly defined user groups, such as depressed inpatients or outpatients with stringently confirmed diagnoses, in order to establish with precision how the program operates among different user groups. A third and related limitation is that the depressed participants in this study may have differed from other depressed adults in that they were more comfortable with computer technology. That is, these participants were recruited in online depression discussion/support groups, so they were presumably relatively experienced computer/Internet users. It remains to be seen whether the effects reported for this group generalize to less computer-literate populations. A fourth potential limitation concerns the program’s lack of multimedia components. Conceivably, the effectiveness of *Deprexis* could be enhanced further by integrating audio or video clips. The downside, though, would be the need for more sophisticated computers and high-speed Internet connections. Follow-up studies are required, then, to examine the processes and components that might further enhance the program’s effectiveness, to delineate the contextual moderators defining the program’s optimal conditions of use, and to understand the mediators explaining how the program’s effects unfold in different user groups (see also Caspar [86] for a discussion of future research directions in this area).

### Conclusion

The present study showed that an integrative online treatment program—*Deprexis*—was effective in improving symptoms of depression among many of its users. On average, program users experienced lasting symptom reductions and improvements in functioning, whereas those who did not use the program remained at their original level of distress and dysfunction. Future studies could examine how the program can best be deployed to reach those who might benefit from its use, how large-scale adoption of the program could help address unmet treatment needs, and how the therapeutic effects achieved by the program unfold on changes at the behavioral, cognitive, interpersonal, and other levels of analysis.

## Multimedia Appendix 1

Deprexis screenshots

## Abbreviations

ACT: acceptance and commitment therapy
ANOVA: analysis of variance
BA: behavioral activation
BDI: Beck Depression Inventory
CBT: cognitive behavioral therapy
IPT: interpersonal psychotherapy
IRB: internal review board
ITT: intention-to-treat
LOCF: last observation carried forward
MBCT: mindfulness-based cognitive therapy
TAU: treatment-as-usual
WSA: work and social adjustment scale
