# Supplementary material for: Effectiveness of a Novel Integrative Online Treatment for Depression (Deprexis): Randomized Controlled Trial
Source: J Med Internet Res. 2009 May 11;11(2):e15. doi: 10.2196/jmir.1151 (PMC2762808; doi:10.2196/jmir.1151)
Supplement: Supplementary file 1 [file jmir_v11i2e15_app1.pdf]

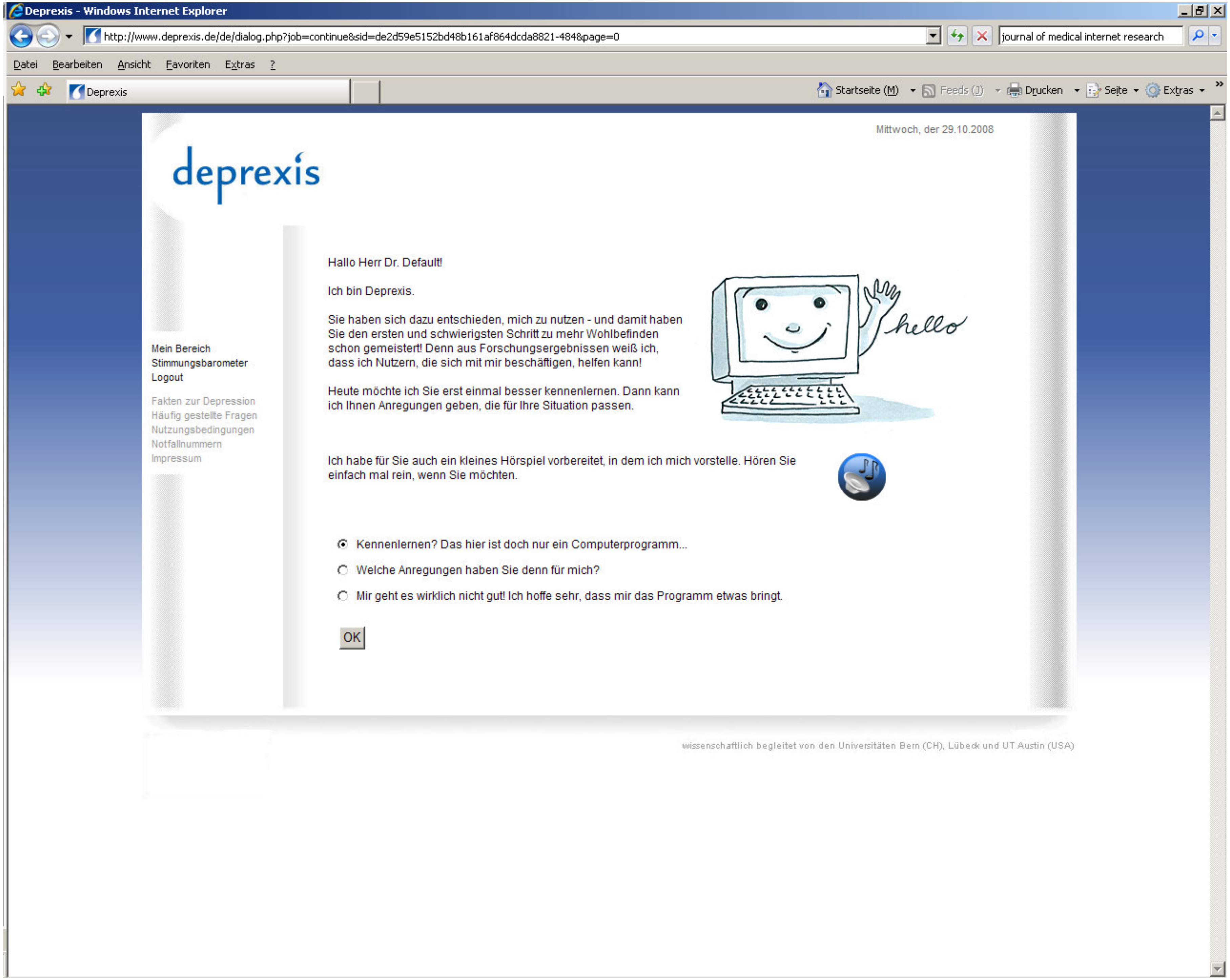
Deprexis - Windows Internet Explorer
http://www.deprexis.de/de/dialog.php?job=continue&sid=de2d59e5152bd48b161af864dcda8821-484&page=0
journal of medical internet research
Datei Bearbeiten Ansicht Favoriten Extras ?
Deprexis
Startseite (M) Feeds (J) Drucken Seite Extras
Mittwoch, der 29.10.2008
deprexis
Hallo Herr Dr. Default!
Ich bin Deprexis.
Sie haben sich dazu entschieden, mich zu nutzen - und damit haben Sie den ersten und schwierigsten Schritt zu mehr Wohlbefinden schon gemeistert! Denn aus Forschungsergebnissen weiß ich, dass ich Nutzern, die sich mit mir beschäftigen, helfen kann!
Heute möchte ich Sie erst einmal besser kennenlernen. Dann kann ich Ihnen Anregungen geben, die für Ihre Situation passen.
hello
Ich habe für Sie auch ein kleines Hörspiel vorbereitet, in dem ich mich vorstelle. Hören Sie einfach mal rein, wenn Sie möchten.
Mein Bereich
Stimmungsbarometer
Logout
Fakten zur Depression
Häufig gestellte Fragen
Nutzungsbedingungen
Notfallnummern
Impressum
Kennenlernen? Das hier ist doch nur ein Computerprogramm...
Welche Anregungen haben Sie denn für mich?
Mir geht es wirklich nicht gut! Ich hoffe sehr, dass mir das Programm etwas bringt.
OK
wissenschaftlich begleitet von den Universitäten Bern (CH), Lübeck und UT Austin (USA)

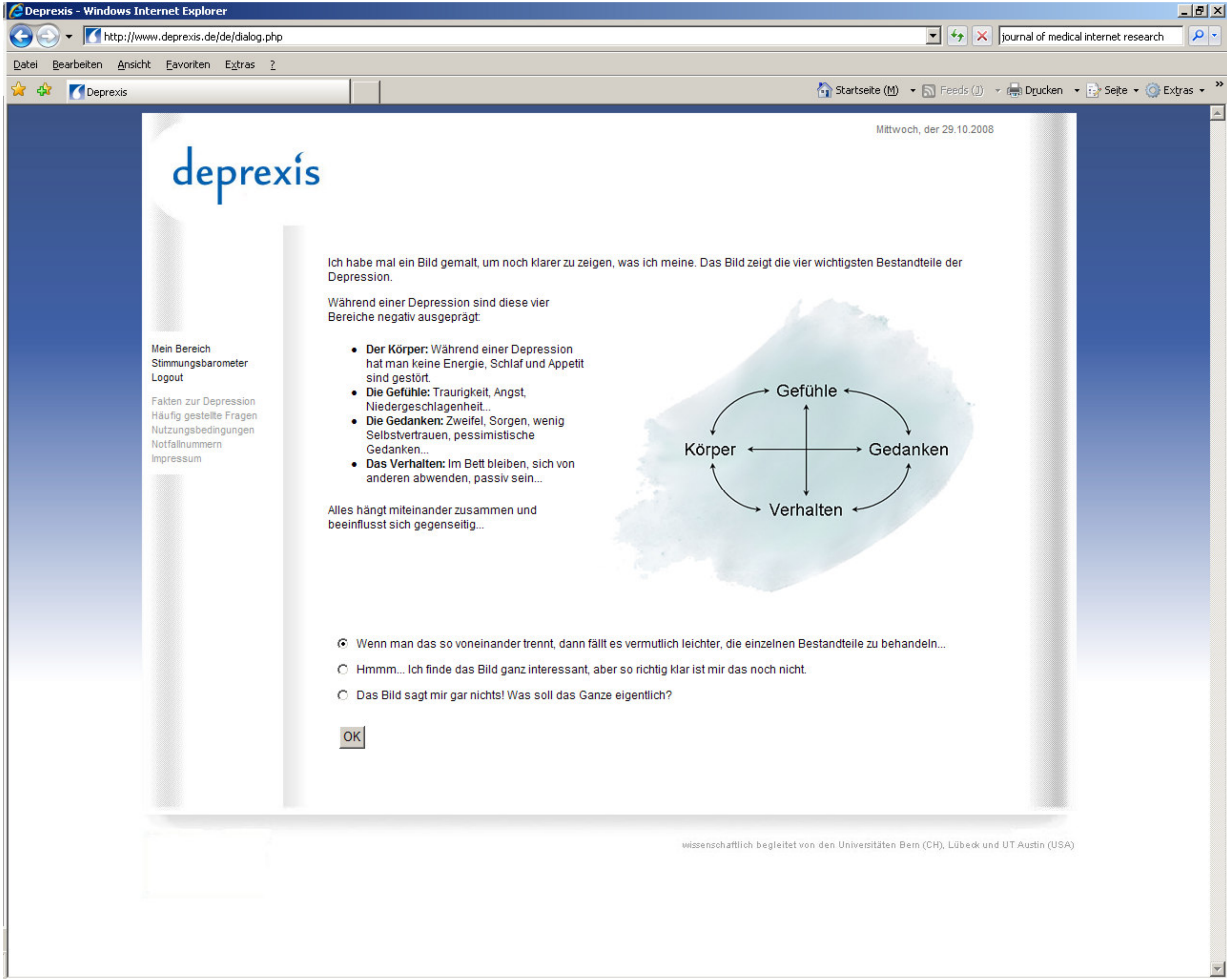
Deprexis - Windows Internet Explorer
http://www.deprexis.de/de/dialog.php
journal of medical internet research
Datei Bearbeiten Ansicht Favoriten Extras ?
Deprexis
Startseite (M) Feeds (J) Drucken Seite Extras
Mittwoch, der 29.10.2008
deprexis
Ich habe mal ein Bild gemalt, um noch klarer zu zeigen, was ich meine. Das Bild zeigt die vier wichtigsten Bestandteile der Depression.
Während einer Depression sind diese vier Bereiche negativ ausgeprägt:
Der Körper: Während einer Depression hat man keine Energie, Schlaf und Appetit sind gestört.
Die Gefühle: Traurigkeit, Angst, Niedergeschlagenheit...
Die Gedanken: Zweifel, Sorgen, wenig Selbstvertrauen, pessimistische Gedanken...
Das Verhalten: Im Bett bleiben, sich von anderen abwenden, passiv sein...
Alles hängt miteinander zusammen und beeinflusst sich gegenseitig...
Gefühle
Körper
Gedanken
Verhalten
Mein Bereich
Stimmungsbarometer
Logout
Fakten zur Depression
Häufig gestellte Fragen
Nutzungsbedingungen
Notfallnummern
Impressum
Wenn man das so voneinander trennt, dann fällt es vermutlich leichter, die einzelnen Bestandteile zu behandeln...
Hmmm... Ich finde das Bild ganz interessant, aber so richtig klar ist mir das noch nicht.
Das Bild sagt mir gar nichts! Was soll das Ganze eigentlich?
OK
wissenschaftlich begleitet von den Universitäten Bern (CH), Lübeck und UT Austin (USA)

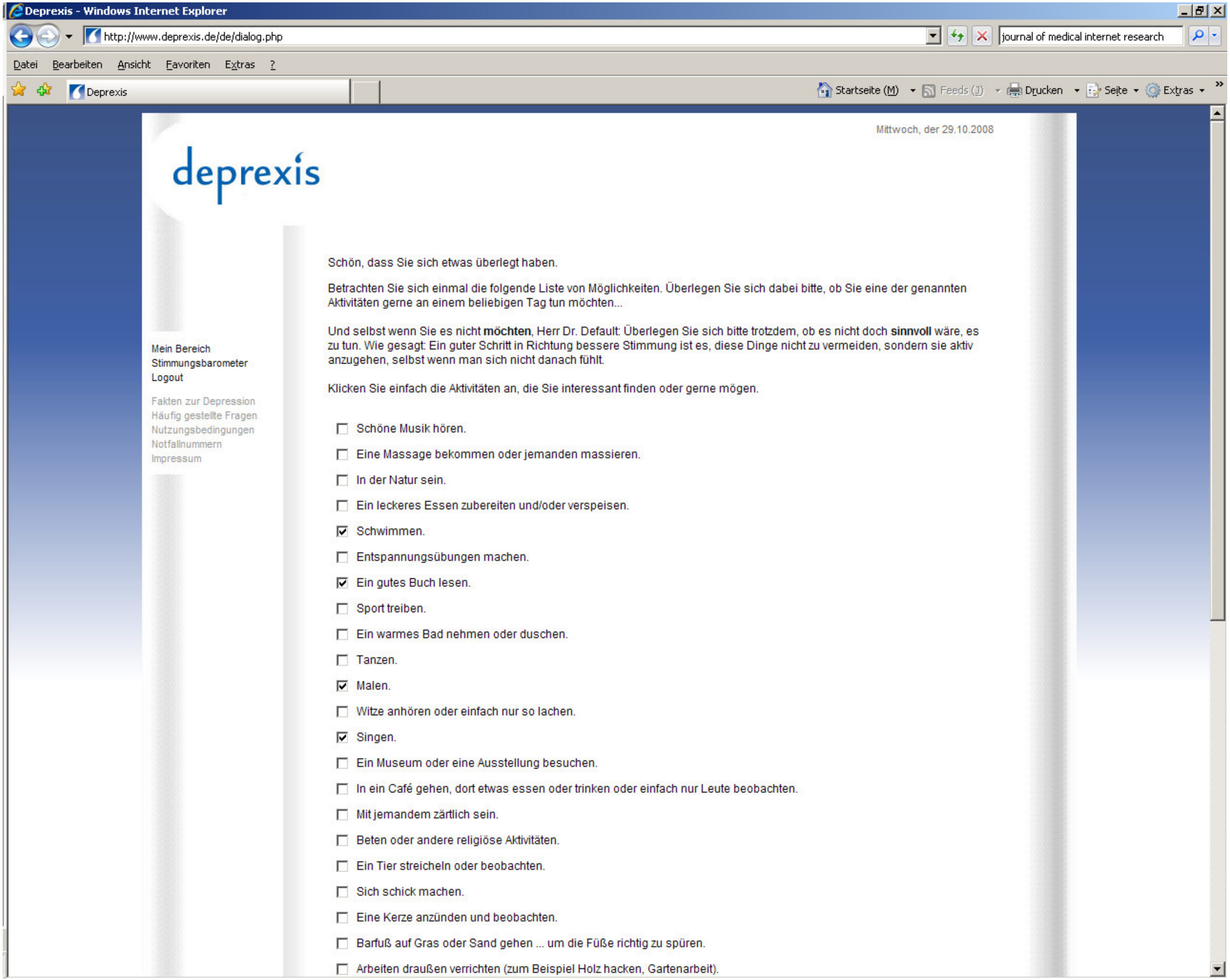
Deprexis - Windows Internet Explorer
http://www.deprexis.de/de/dialog.php
journal of medical internet research
Datei Bearbeiten Ansicht Favoriten Extras ?
Deprexis
Startseite (M) Feeds (J) Drucken Seite Extras
Mittwoch, der 29.10.2008
deprexis
Mein Bereich
Stimmungsbarometer
Logout
Fakten zur Depression
Häufig gestellte Fragen
Nutzungsbedingungen
Notfallnummern
Impressum
Schön, dass Sie sich etwas überlegt haben.
Betrachten Sie sich einmal die folgende Liste von Möglichkeiten. Überlegen Sie sich dabei bitte, ob Sie eine der genannten Aktivitäten gerne an einem beliebigen Tag tun möchten...
Und selbst wenn Sie es nicht möchten, Herr Dr. Default: Überlegen Sie sich bitte trotzdem, ob es nicht doch sinnvoll wäre, es zu tun. Wie gesagt: Ein guter Schritt in Richtung bessere Stimmung ist es, diese Dinge nicht zu vermeiden, sondern sie aktiv anzugehen, selbst wenn man sich nicht danach fühlt.
Klicken Sie einfach die Aktivitäten an, die Sie interessant finden oder gerne mögen.
Schöne Musik hören.
Eine Massage bekommen oder jemanden massieren.
In der Natur sein.
Ein leckeres Essen zubereiten und/oder verspeisen.
Schwimmen.
Entspannungsübungen machen.
Ein gutes Buch lesen.
Sport treiben.
Ein warmes Bad nehmen oder duschen.
Tanzen.
Malen.
Witze anhören oder einfach nur so lachen.
Singen.
Ein Museum oder eine Ausstellung besuchen.
In ein Café gehen, dort etwas essen oder trinken oder einfach nur Leute beobachten.
Mit jemandem zärtlich sein.
Beten oder andere religiöse Aktivitäten.
Ein Tier streicheln oder beobachten.
Sich schick machen.
Eine Kerze anzünden und beobachten.
Barfuß auf Gras oder Sand gehen ... um die Füße richtig zu spüren.
Arbeiten draußen verrichten (zum Beispiel Holz hacken, Gartenarbeit).

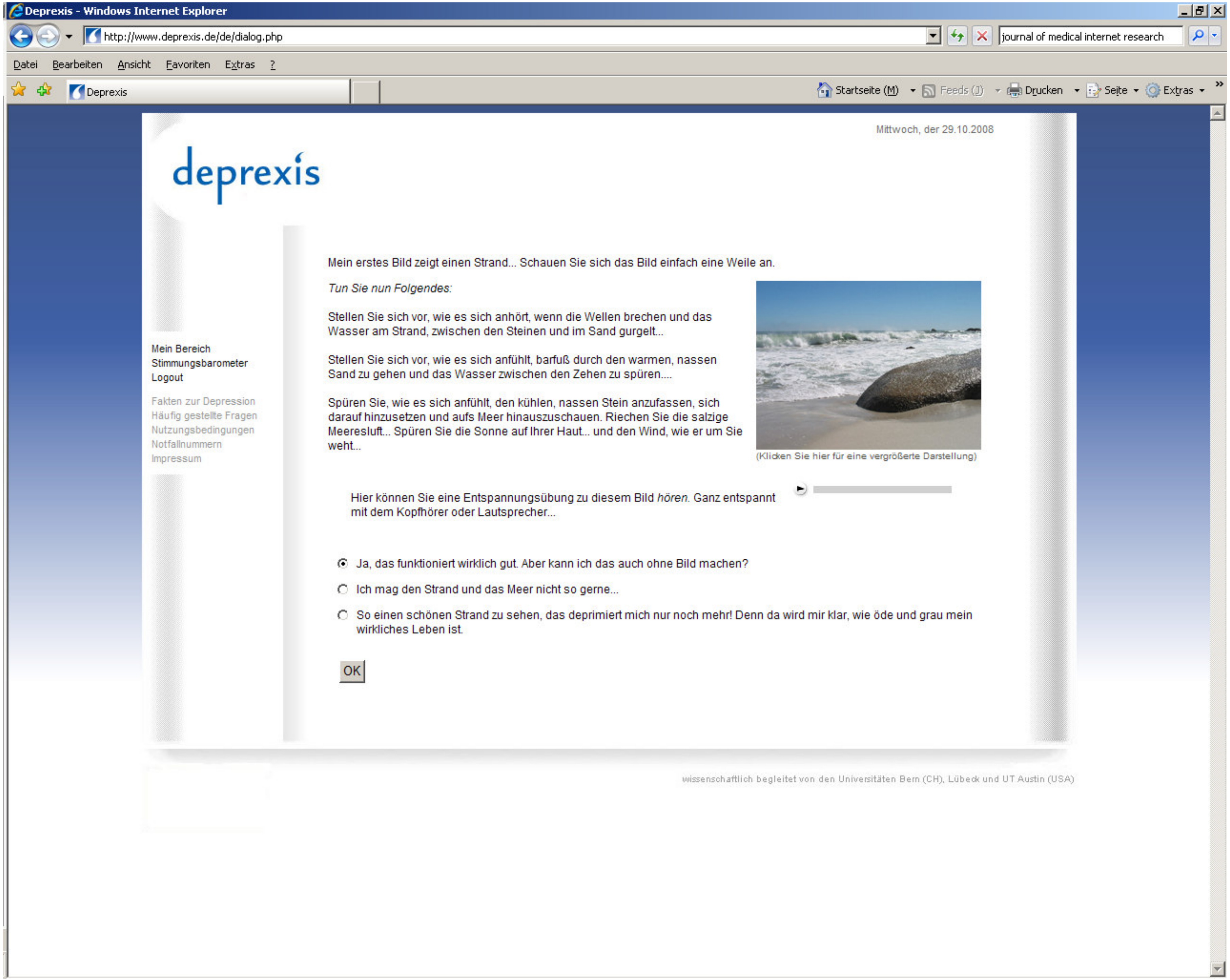
Deprexis - Windows Internet Explorer
http://www.deprexis.de/de/dialog.php
journal of medical internet research
Datei Bearbeiten Ansicht Favoriten Extras ?
Deprexis
Startseite (M) Feeds (J) Drucken Seite Extras
Mittwoch, der 29.10.2008
deprexis
Mein erstes Bild zeigt einen Strand... Schauen Sie sich das Bild einfach eine Weile an.
Tun Sie nun Folgendes:
Stellen Sie sich vor, wie es sich anhört, wenn die Wellen brechen und das Wasser am Strand, zwischen den Steinen und im Sand gurgelt...
Stellen Sie sich vor, wie es sich anfühlt, barfuß durch den warmen, nassen Sand zu gehen und das Wasser zwischen den Zehen zu spüren....
Spüren Sie, wie es sich anfühlt, den kühlen, nassen Stein anzufassen, sich darauf hinzusetzen und aufs Meer hinauszuschauen. Riechen Sie die salzige Meeresluft... Spüren Sie die Sonne auf Ihrer Haut... und den Wind, wie er um Sie weht...
(Klicken Sie hier für eine vergrößerte Darstellung)
Mein Bereich
Stimmungsbarometer
Logout
Fakten zur Depression
Häufig gestellte Fragen
Nutzungsbedingungen
Notfallnummern
Impressum
Hier können Sie eine Entspannungsübung zu diesem Bild hören. Ganz entspannt mit dem Kopfhörer oder Lautsprecher...
Ja, das funktioniert wirklich gut. Aber kann ich das auch ohne Bild machen?
Ich mag den Strand und das Meer nicht so gerne...
So einen schönen Strand zu sehen, das deprimiert mich nur noch mehr! Denn da wird mir klar, wie öde und grau mein wirkliches Leben ist.
OK
wissenschaftlich begleitet von den Universitäten Bern (CH), Lübeck und UT Austin (USA)

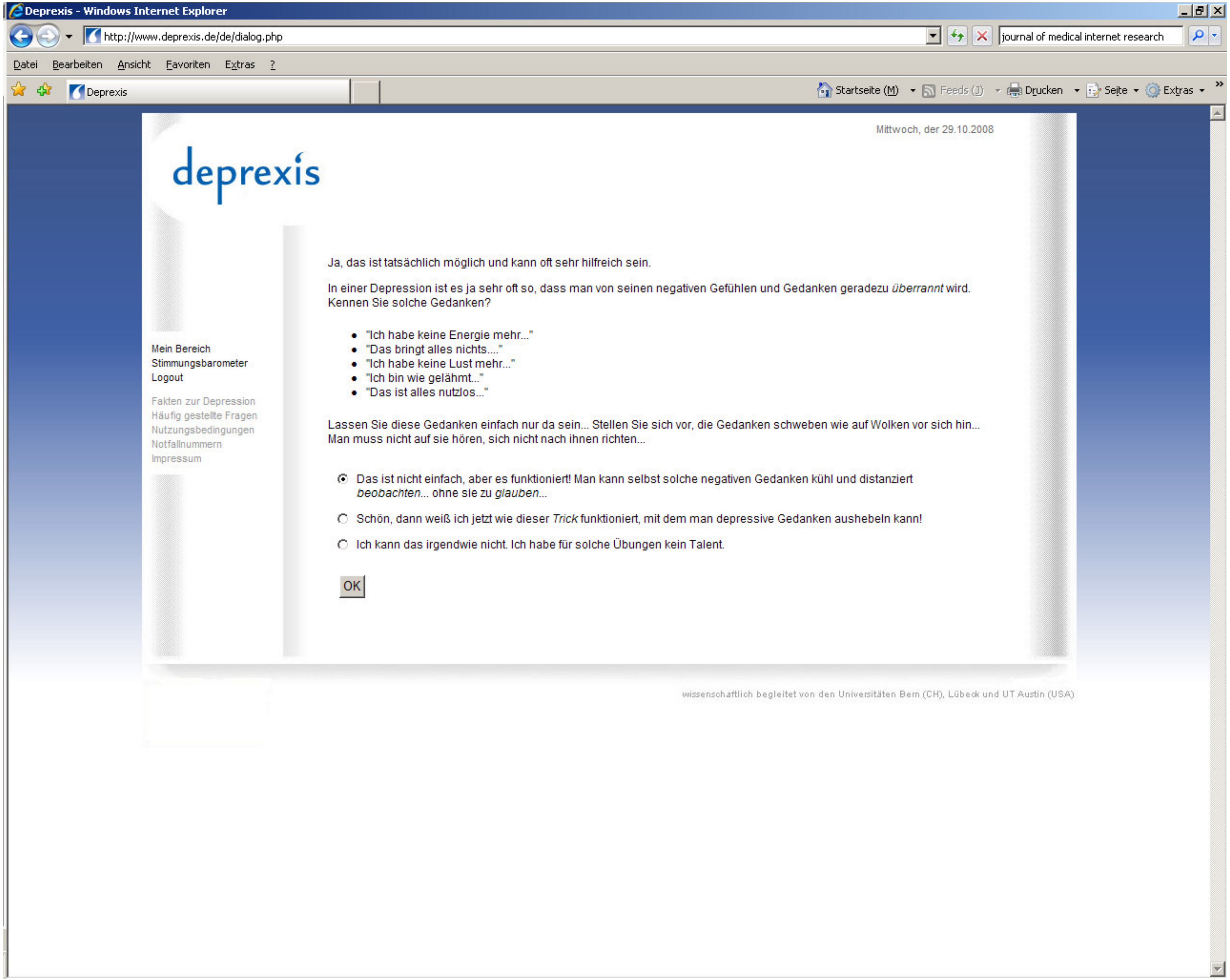
Deprexis - Windows Internet Explorer
http://www.deprexis.de/de/dialog.php
journal of medical internet research
Datei Bearbeiten Ansicht Favoriten Extras ?
Deprexis
Startseite (M) Feeds (J) Drucken Seite Extras
Mittwoch, der 29.10.2008
deprexis
Mein Bereich
Stimmungsbarometer
Logout
Fakten zur Depression
Häufig gestellte Fragen
Nutzungsbedingungen
Notfallnummern
Impressum
Ja, das ist tatsächlich möglich und kann oft sehr hilfreich sein.
In einer Depression ist es ja sehr oft so, dass man von seinen negativen Gefühlen und Gedanken geradezu überrannt wird. Kennen Sie solche Gedanken?
"Ich habe keine Energie mehr..."
"Das bringt alles nichts...."
"Ich habe keine Lust mehr..."
"Ich bin wie gelähmt..."
"Das ist alles nutzlos..."
Lassen Sie diese Gedanken einfach nur da sein... Stellen Sie sich vor, die Gedanken schweben wie auf Wolken vor sich hin... Man muss nicht auf sie hören, sich nicht nach ihnen richten...
Das ist nicht einfach, aber es funktioniert! Man kann selbst solche negativen Gedanken kühl und distanziert beobachten... ohne sie zu glauben...
Schön, dann weiß ich jetzt wie dieser Trick funktioniert, mit dem man depressive Gedanken aushebeln kann!
Ich kann das irgendwie nicht. Ich habe für solche Übungen kein Talent.
OK
wissenschaftlich begleitet von den Universitäten Bern (CH), Lübeck und UT Austin (USA)

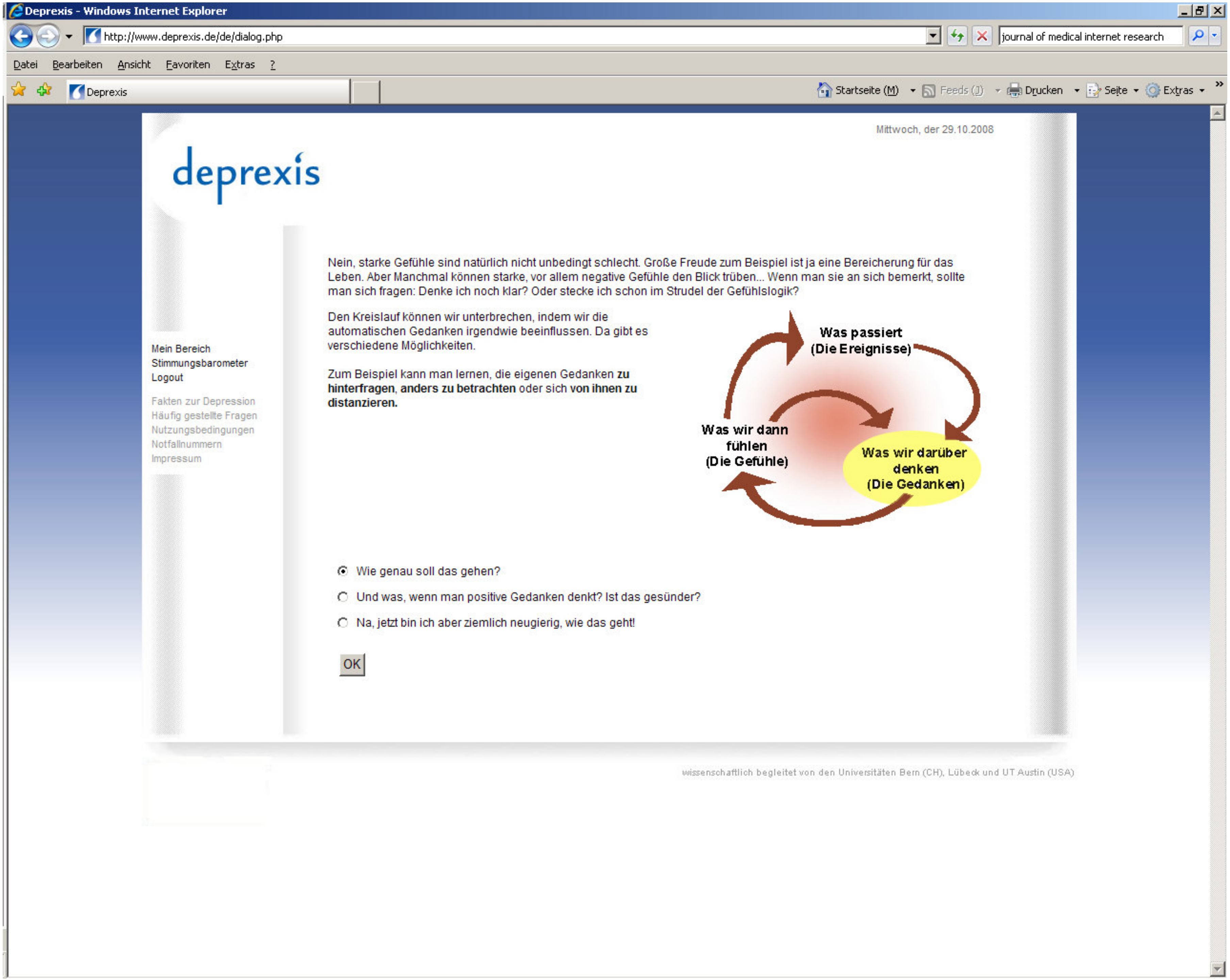
Deprexis - Windows Internet Explorer
http://www.deprexis.de/de/dialog.php
journal of medical internet research
Datei Bearbeiten Ansicht Favoriten Extras ?
Deprexis
Startseite (M) Feeds (J) Drucken Seite Extras
Mittwoch, der 29.10.2008
deprexis
Nein, starke Gefühle sind natürlich nicht unbedingt schlecht. Große Freude zum Beispiel ist ja eine Bereicherung für das Leben. Aber Manchmal können starke, vor allem negative Gefühle den Blick trüben... Wenn man sie an sich bemerkt, sollte man sich fragen: Denke ich noch klar? Oder stecke ich schon im Strudel der Gefühlslogik?
Den Kreislauf können wir unterbrechen, indem wir die automatischen Gedanken irgendwie beeinflussen. Da gibt es verschiedene Möglichkeiten.
Zum Beispiel kann man lernen, die eigenen Gedanken zu hinterfragen, anders zu betrachten oder sich von ihnen zu distanzieren.
Was passiert (Die Ereignisse)
Was wir darüber denken (Die Gedanken)
Was wir dann fühlen (Die Gefühle)
Mein Bereich
Stimmungsbarometer
Logout
Fakten zur Depression
Häufig gestellte Fragen
Nutzungsbedingungen
Notfallnummern
Impressum
Wie genau soll das gehen?
Und was, wenn man positive Gedanken denkt? Ist das gesünder?
Na, jetzt bin ich aber ziemlich neugierig, wie das geht!
OK
wissenschaftlich begleitet von den Universitäten Bern (CH), Lübeck und UT Austin (USA)

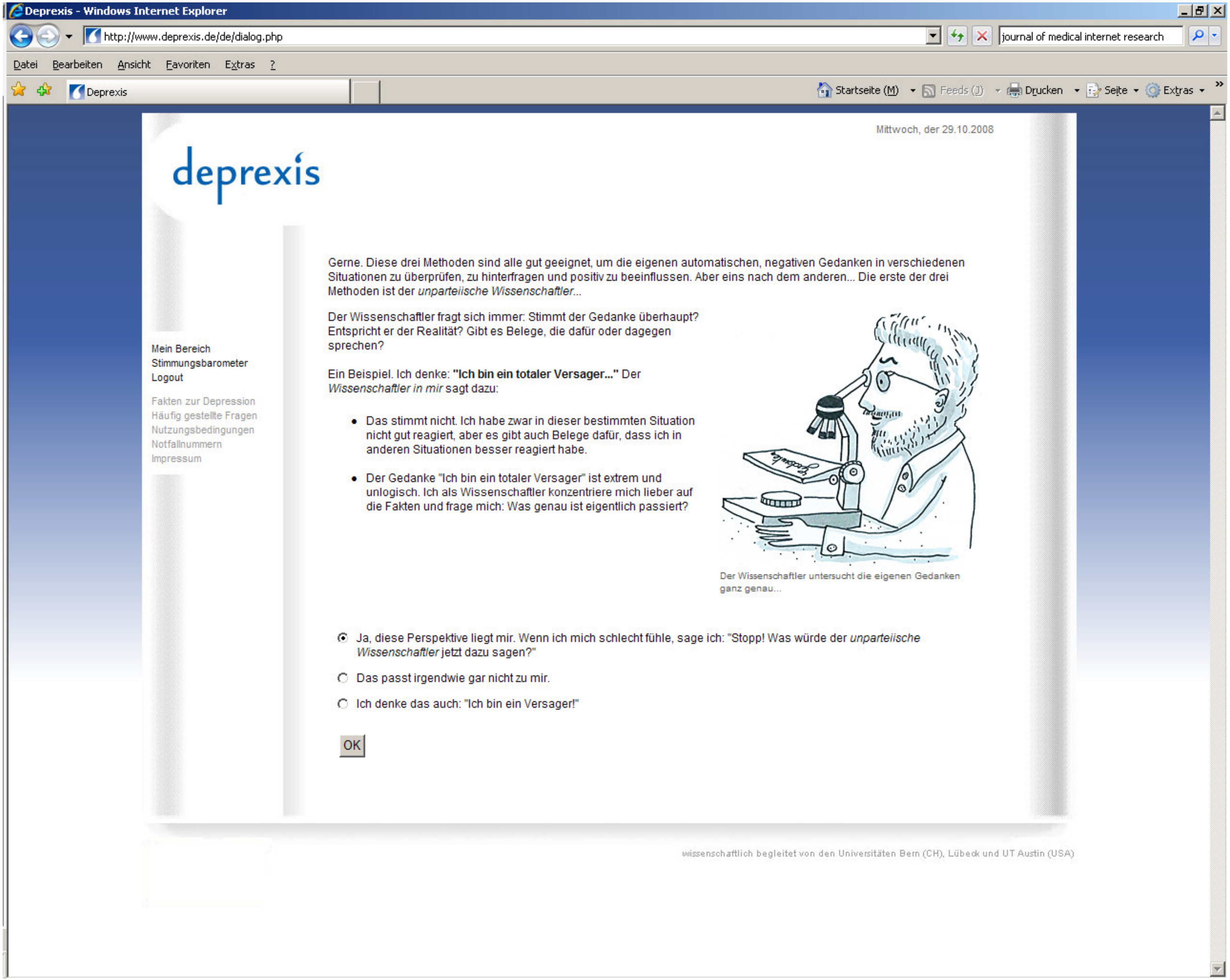
Deprexis - Windows Internet Explorer
http://www.deprexis.de/de/dialog.php
journal of medical internet research
Datei Bearbeiten Ansicht Favoriten Extras ?
Deprexis
Startseite (M) Feeds (J) Drucken Seite Extras
Mittwoch, der 29.10.2008
deprexis
Gerne. Diese drei Methoden sind alle gut geeignet, um die eigenen automatischen, negativen Gedanken in verschiedenen Situationen zu überprüfen, zu hinterfragen und positiv zu beeinflussen. Aber eins nach dem anderen... Die erste der drei Methoden ist der unparteiische Wissenschaftler...
Der Wissenschaftler fragt sich immer: Stimmt der Gedanke überhaupt? Entspricht er der Realität? Gibt es Belege, die dafür oder dagegen sprechen?
Ein Beispiel. Ich denke: "Ich bin ein totaler Versager..." Der Wissenschaftler in mir sagt dazu:
Das stimmt nicht. Ich habe zwar in dieser bestimmten Situation nicht gut reagiert, aber es gibt auch Belege dafür, dass ich in anderen Situationen besser reagiert habe.
Der Gedanke "Ich bin ein totaler Versager" ist extrem und unlogisch. Ich als Wissenschaftler konzentriere mich lieber auf die Fakten und frage mich: Was genau ist eigentlich passiert?
Der Wissenschaftler untersucht die eigenen Gedanken ganz genau...
Mein Bereich
Stimmungsbarometer
Logout
Fakten zur Depression
Häufig gestellte Fragen
Nutzungsbedingungen
Notfallnummern
Impressum
Ja, diese Perspektive liegt mir. Wenn ich mich schlecht fühle, sage ich: "Stopp! Was würde der unparteiische Wissenschaftler jetzt dazu sagen?"
Das passt irgendwie gar nicht zu mir.
Ich denke das auch: "Ich bin ein Versager!"
OK
wissenschaftlich begleitet von den Universitäten Bern (CH), Lübeck und UT Austin (USA)

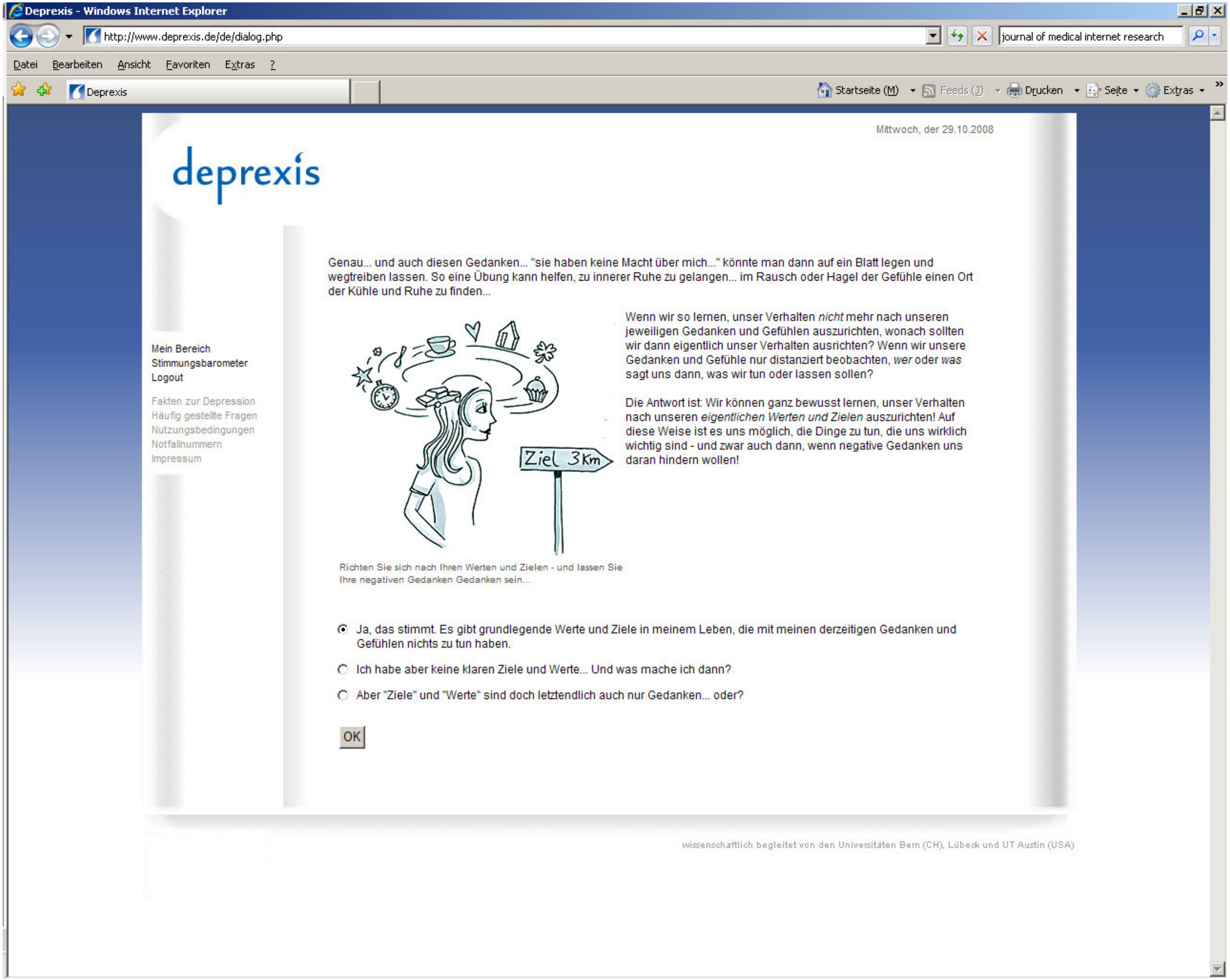
Deprexis - Windows Internet Explorer
http://www.deprexis.de/de/dialog.php
journal of medical internet research
Datei Bearbeiten Ansicht Favoriten Extras ?
Deprexis
Startseite (M) Feeds (J) Drucken Seite Extras
Mittwoch, der 29.10.2008
deprexis
Genau... und auch diesen Gedanken... "sie haben keine Macht über mich..." könnte man dann auf ein Blatt legen und wegtreiben lassen. So eine Übung kann helfen, zu innerer Ruhe zu gelangen... im Rausch oder Hagel der Gefühle einen Ort der Kühle und Ruhe zu finden...
Mein Bereich
Stimmungsbarometer
Logout
Fakten zur Depression
Häufig gestellte Fragen
Nutzungsbedingungen
Notfallnummern
Impressum
Ziel 3Km
Richten Sie sich nach Ihren Werten und Zielen - und lassen Sie Ihre negativen Gedanken Gedanken sein...
Wenn wir so lernen, unser Verhalten nicht mehr nach unseren jeweiligen Gedanken und Gefühlen auszurichten, wonach sollten wir dann eigentlich unser Verhalten ausrichten? Wenn wir unsere Gedanken und Gefühle nur distanziert beobachten, wer oder was sagt uns dann, was wir tun oder lassen sollen?
Die Antwort ist: Wir können ganz bewusst lernen, unser Verhalten nach unseren eigentlichen Werten und Zielen auszurichten! Auf diese Weise ist es uns möglich, die Dinge zu tun, die uns wirklich wichtig sind - und zwar auch dann, wenn negative Gedanken uns daran hindern wollen!
Ja, das stimmt. Es gibt grundlegende Werte und Ziele in meinem Leben, die mit meinen derzeitigen Gedanken und Gefühlen nichts zu tun haben.
Ich habe aber keine klaren Ziele und Werte... Und was mache ich dann?
Aber "Ziele" und "Werte" sind doch letztendlich auch nur Gedanken... oder?
OK
wissenschaftlich begleitet von den Universitäten Bern (CH), Lübeck und UT Austin (USA)

Deprexis - Windows Internet Explorer

http://www.deprexis.de/de/dialog.php

journal of medical internet research

Datei Bearbeiten Ansicht Favoriten Extras ?

Deprexis

Startseite (M) Feeds (J) Drucken Seite Extras

Mittwoch, der 29.10.2008

deprexis

Mein Bereich
Stimmungsbarometer
Logout

Fakten zur Depression
Häufig gestellte Fragen
Nutzungsbedingungen
Notfallnummern
Impressum

Ja, das Erkennen und Beschreiben des Problems ist ein wichtiger erster Schritt.

Das Aufschreiben hat verschiedene Vorteile:

- Indem man das Problem genau beschreibt, bringt man es aus seinem Kopf heraus - aufs Papier. Das schafft Abstand und kann befreiend wirken!
- Verworrene Grübeleien lassen sich durch das Aufschreiben ordnen.
- So sieht man leichter, wo man den Hebel ansetzen kann, um das Problem zu lösen.
- Man kann das Geschriebene leichter mit anderen besprechen.

Aufschreiben ist ein sinnvoller erster Schritt - **besonders wenn man das Problem in konkrete, präzise Worte fasst anstatt es vage oder oberflächlich zu beschreiben.**

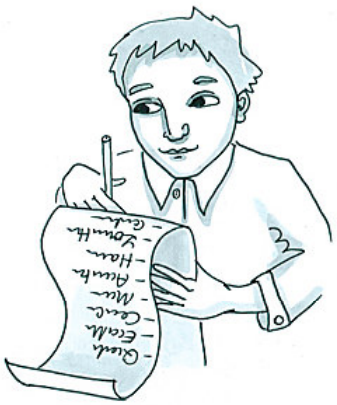

Das hilft... Durch Aufschreiben sehe ich klarer!

- (•) Können Sie mir ein Beispiel geben, wie man das am besten macht?
- ( ) Wie soll das Aufschreiben Abstand schaffen? Das Problem ist ja trotzdem noch da!
- ( ) Das habe ich verstanden. Und was ist dann der nächste Schritt?
- ( ) Welche Schritte kommen eigentlich noch in Ihrem Plan? Eine Übersicht wäre schön...

OK

wissenschaftlich begleitet von den Universitäten Bern (CH), Lübeck und UT Austin (USA)

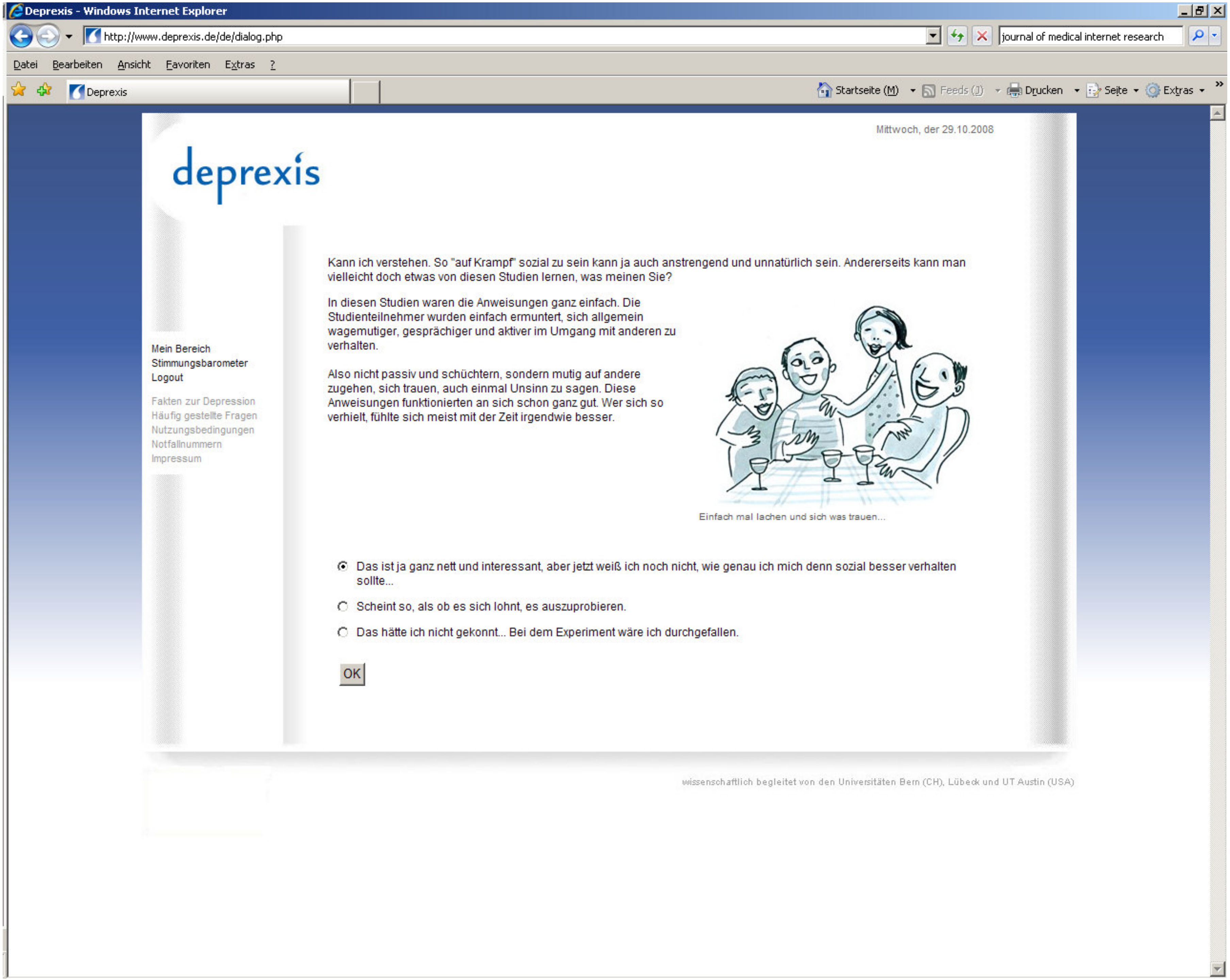
Deprexis - Windows Internet Explorer
http://www.deprexis.de/de/dialog.php
journal of medical internet research
Datei Bearbeiten Ansicht Favoriten Extras ?
Deprexis
Startseite (M) Feeds (J) Drucken Seite Extras
Mittwoch, der 29.10.2008
deprexis
Mein Bereich
Stimmungsbarometer
Logout
Fakten zur Depression
Häufig gestellte Fragen
Nutzungsbedingungen
Notfallnummern
Impressum
Kann ich verstehen. So "auf Krampf" sozial zu sein kann ja auch anstrengend und unnatürlich sein. Andererseits kann man vielleicht doch etwas von diesen Studien lernen, was meinen Sie?
In diesen Studien waren die Anweisungen ganz einfach. Die Studienteilnehmer wurden einfach ermuntert, sich allgemein wagemutiger, gesprächiger und aktiver im Umgang mit anderen zu verhalten.
Also nicht passiv und schüchtern, sondern mutig auf andere zugehen, sich trauen, auch einmal Unsinn zu sagen. Diese Anweisungen funktionierten an sich schon ganz gut. Wer sich so verhielt, fühlte sich meist mit der Zeit irgendwie besser.
Einfach mal lachen und sich was trauen...
Das ist ja ganz nett und interessant, aber jetzt weiß ich noch nicht, wie genau ich mich denn sozial besser verhalten sollte...
Scheint so, als ob es sich lohnt, es auszuprobieren.
Das hätte ich nicht gekonnt... Bei dem Experiment wäre ich durchgefallen.
OK
wissenschaftlich begleitet von den Universitäten Bern (CH), Lübeck und UT Austin (USA)
